# A special report of current state of the medical physicist workforce — results of the 2012 ASTRO Comprehensive Workforce Study

**DOI:** 10.1120/jacmp.v16i3.5232

**Published:** 2015-05-08

**Authors:** Erli Chen, Anna Arnone, Jussi K Sillanpaa, Yan Yu, Michael D. Mills

**Affiliations:** ^1^ Radiation Oncology Department Cheshire Medical Center Keene NH; ^2^ Member Relations and Communications American Society for Radiation Oncology Fairfax VA; ^3^ Department of Radiation Oncology Kaiser Permanente San Francisco CA; ^4^ Radiation Oncology Department Thomas Jefferson University Philadelphia PA; ^5^ Department of Radiation Oncology University of Louisville School of Medicine Louisville KY USA

**Keywords:** ASTRO Comprehensive Workforce Study, medical physicist workforce

## Abstract

The medical physics profession is undergoing significant changes. Starting in 2014, candidates registering for certification exams by the American Board of Radiology must have completed a CAMPEP‐accredited residency. This requirement, along with tightened state regulations, uncertainty in future reimbursement, and a stronger emphasis on board certification, have raised questions concerning the state of the medical physics workforce and its ability to adapt to changing requirements. In 2012, ASTRO conducted a workforce study of the comprehensive field of radiation oncology. This article reviews the findings of the medical physics section of the study, including age and gender distribution, educational background, workload, and primary work setting. We also report on job satisfaction, the perceived supply and demand of medical physicists, and the medical physicists' main concerns pertaining to patient safety and quality assurance.

PACS number: 87.90

## I. INTRODUCTION

The professional landscape of radiation oncology is undergoing significant changes. Although the size and average age of the United States population continue to increase, resulting in a greater number of cancer patients, the future reimbursement of radiation oncology services is hard to predict.^(1)^ The profession of medical physics faces additional uncertainties, mainly connected to board certification. Starting in 2014, candidates registering for certification exams by the American Board of Radiology must have completed a Commission on Accreditation of Medical Physics Educational Programs, Inc. (CAMPEP) accredited residency. The number of such residencies, although increasing, is still below the projected annual need of medical physicists.^(2)^ There have also been recent changes in Nuclear Regulatory Commission (NRC) and state regulations (e.g., state licensure of physicists in some states) and a renewed emphasis on patient safety. These challenges, together with the fast pace of technological change in the field, have raised questions about the state of the medical physics workforce and its ability to adapt to changing requirements.

To help inform these issues, the American Society for Radiation Oncology (ASTRO) commissioned a workforce study of the field of radiation oncology, including radiation oncology medical physicists. The study was conducted in collaboration with other professional specialty societies, including the American Association of Physicists in Medicine (AAPM). In this paper, we report on the age and gender distribution, educational background, workload, and primary work setting of the radiation oncology medical physics workforce, along with data on job satisfaction, the perceived supply and demand of medical physicists, and the thoughts of practicing medical physicists on improving quality and safety in patient care.

## II. MATERIALS AND METHODS

The data for this study was collected using an Internet‐based survey. Details of study methodology can be found in “An Assessment of the Current US Radiation Oncology Workforce: Methodology and Global Results of the American Society for Radiation Oncology 2012 Workforce Study”.^(3)^ The medical physicist (MP) segment of the survey was limited to physicists practicing in the United States. The survey questions included multiple choice, Likert scale, and open‐ended questions. An expert panel, made up of volunteers from the medical physics workforce segment and representatives of the AAPM, was assembled to evaluate the survey questions. Through a series of conference calls, questions relevant and specific to the medical physics workforce segment were developed. The draft survey was also reviewed by the ASTRO Workforce Subcommittee and the ASTRO Board of Directors. The questions were then pilot‐tested using cognitive response testing in order to ensure that respondents consistently understood the questions in the way in which they were intended. The final survey questions were compiled in Qualtrics, an online survey software program.^(4)^ IBM SPSS Statistics version 205 was used to analyze the data.

Personalized emails with a link to the survey were sent to MP members of AAPM and/or ASTRO. Membership lists were cross‐matched to prevent duplicate invitations and responses. The survey opened on January 12, 2012. Reminders were sent at one, two, and three weeks postlaunch, and at the end of the survey period. AAPM sent reminders in addition to ASTRO. The data collection period was approximately three months and the survey closed on April 13, 2012.

## III. RESULTS & DISCUSSION

A total of 35,204 surveys were sent out to all segments of the workforce; with 6,765 completed surveys returned, the overall response rate was 19%. A total of 6,286 surveys were sent to medical physicists (MPs), with a response rate of 18% (1,105).

### A. Demographics and practice characteristics

California, New York, Texas, Florida, and Pennsylvania are the states in which the highest numbers of MP respondents practice. When describing the type of community in which their practice is located, 54.8% report urban, 33.5% suburban, and 11.8% rural setting. Ninety‐four percent of MPs work full‐time, 4% part time, 1% as locum tenens, and 1% were not currently working at the time of the survey but were looking for a new position. The mean number of years of work experience was 16, with 50% of MPs indicating that they obtained their radiation oncology‐specific training on‐the‐job. Sixty‐four percent of respondents reported having ABR certification (31.7% Lifetime and 32.7% Time Limited). Among the respondents, 76% were white, 19% Asian, 5% Indian, 2% African American, and 1% other. (Note: respondents were allowed to self‐identify in more than one category.) The mean age of the MP was 46.6 years, with 19% less than 35 years of age, 25% between 35 and 44, 28% between 45 and 54, 22% between 55 and 64, and 6% age 65 or older. Seventy‐seven percent of respondents were male. The greatest number of MPs (40.8%) reported working in a hospital‐based setting, while 33.7% reported working in private practice, and 25.5% reported working in an academic setting. An average of 4.9 full‐time physicists are employed in the academic setting, 4.2 in private practice, and 2.6 in hospital based (Table 1). There are significantly more FTE MPs in the academic/university setting compared with other settings. The average number of hours worked per week is 48. Table 1 also shows patient load, practice size, and services provided.

**Table 1:** Medical physicist data based on practice setting.

	*Academic*	*Hospital Employed*	*Private/Group Practice*
Medical Physicist practice setting (%)	25.5	40.8	33.7
Patients on treatment	137	83	128
New patient consults in 2011	237	253	263
Practice size (# of Medical Physicists)	4.9	2.6	4.2
Patients on treatment per MP	28	32	30
Provides SRS, SBRT (%)	85	69	60
Provides HDR Brachytherapy (%)	70	65	60
Provides IORT (%)	30	8	6
Provides Proton Therapy (%)	15	1	2

### B. Supply and demand

**Figure 1:**
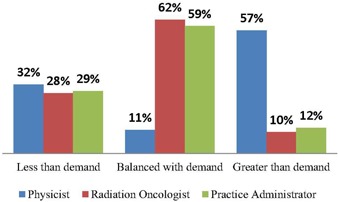
Perception of current supply vs. demand.

Sixty‐two percent of MPs have been with their current practice/employer since at least the beginning of 2008. A slight majority (51.7%) reported not having actively searched for employment within the past three years. Of those who did search for employment, 52.3% said it was difficult to find a position with which they were satisfied. The lack of positions in the desired area and the overall lack of positions were cited as the top two reasons for the difficulty experienced in finding a position. While 57.2% of MPs said that the current supply of medical physicists in their region was greater than demand, only 10% of radiation oncologists and 12% of administrators shared this perception, as shown in Fig. 1. Nearly three‐quarters (71.5%) of MPs reported not having any current vacancies at their workplace, 11.4% reported there were no vacancies but added they were understaffed, and 17.1% reported one or more vacancies. Those reporting vacancies said the Number 1 reason for the vacancy was because the MP resigned (57.2%). Other reasons were growth in practice (35.6%), retirement (3.3%), termination (1.1%), and other (2.8%).

Concerning MP respondents, 42.8% said the personnel shortage would not impact the quality of patient care, including delays and additional time between consultation and treatment start time; 36.1% said it would have a slight negative impact, 13.3% moderately negative impact, 6.7% significant negative impact, and 1.2% said a positive impact.

### C. Satisfaction

Overall, respondents expressed satisfaction with their career as a medical physicist and with their current position. The majority said they often felt a sense of accomplishment at the end of the day and that working as a medical physicist was rewarding. Forty‐two percent of physicists described the overall workload in their current job as heavy, while 39% described the workload as about right. Forty percent reported occasionally feeling burned out by their work (Table 2).

**Table 2:** Satisfaction.

	*Very Satisfied*	*Satisfied*	*Neutral*	*Dissatisfied*	*Very Dissatisfied*
Time available for family and personal life	3.8	34.3	21.9	31.7	8.2
Opportunity to teach MP students	3.3	20.3	54.5	18.6	3.3
Compensation/ salary	7.5	42.4	25.4	20.0	4.7
Staffing resources available	2.9	32.3	32.9	27.4	4.5
Volume of patient load	1.9	38.1	38.8	18.8	2.3
Time spent with patient/tx file	2.3	46.2	38.5	12.1	0.8

### D. Patient safety and quality assurance

Essential responsibilities of a medical physicist's job are ensuring the safety of patients and quality of their radiation treatment. A major component of this is developing and implementing new technology. In order to have a full picture of the current medical physics practice pattern, the survey presented the respondents with a series of statements, to which they were asked to indicate their level of agreement (strongly agree / agree / neither agree or disagree / disagree / strongly disagree). Open‐ended questions about such issues as working hours and the type of medical errors encountered, were also asked.

#### D.1. The distribution of working hours

Medical physicists reported 49% of their time is devoted to patient‐specific clinical tasks, 22% to quality assurance, 6% to research, 10% to administrative tasks, 7% to radiation safety, and 5% to teaching and training.

#### D.2. Ensuring quality and safety

Most MPs (84.5%) reported that their facility required a second independent check on all treatment plans before the first treatment. Out of the MP respondents, 86.7% reported using electronic medical records (the most popular program was MOSAIQ(^6^) (60.9%), followed by ARIA(^7^) (43.5%)). A majority of medical physicists (66.7%) felt that extensive/direct supervision was required for newly hired MP graduates.

#### D.3. Confidence in commissioning and implementing new technology

Overall, MPs are confident when implementing new technology. Those who reported no confidence said it was due to insufficient time or manpower for adequate training. Over half of MPs (56.2%) disagreed or strongly disagreed with the statement that manufacturers' training programs are completely adequate to train physicists and others to deliver therapy with high quality and safety, and 47.8% of respondents agree or strongly agree that there are too few manufacturer‐supported continuing education forums that offer clinically useful material for new and emerging technologies. However, 38.1% of respondents agree or strongly agree that, when new special procedures are introduced into the community, adequate training of sufficient depth and quality is provided at targeted forums led by expert early adopters and users. Out of the MP respondents, 51.9% felt that there isn't a need to create subspecialty certification in radiation oncology physics. The most prevalent reason cited for subspecialty certification not being warranted was practitioners already have adequate training/certification to use/adapt to all treatments (45.7%). Among the 48.1% favoring the creation of subspecialty certification, the most frequently mentioned categories were SRS/SBRT (65.3%), brachytherapy (43.4%), and proton therapy (13.2%).

#### D.4. Medical errors

**Figure 2:**
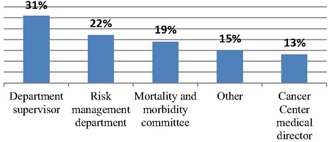
Who is responsible for error follow‐up.

A majority of MP respondents (63.4%) agreed or strongly agreed that, in spite of the complexity associated with IMRT, IGRT, adaptive therapy, segmentation, and gating technologies, the Medical Event is still a valuable concept to elucidate whether radiation therapy was performed improperly. The respondents were asked to identify significant causes of errors in radiation oncology. The percentage agreeing or strongly agreeing was highest for user error (90.9%), followed by substantial increase in complexity of technology (79.1%) and inadequate time for careful checks (76.1%). Eighty‐five percent of respondents reported that their practice/department tracked errors (see Fig. 2 for the entity responsible for follow‐up). Out of the MP respondents, 85.7% would like to participate in a National Safety/Quality database to track errors, if one were available.

#### D.5. Improving patient safety and quality of care

A majority of MPs (73.6%) agree or strongly agree that physicists, dosimetrists, radiation oncologists, and therapists work together with excellent communication, and 70.8% agree or strongly agree that when commissioning new special procedures, a peer review by a qualified medical physicist familiar with the procedure should be standard practice in the community. Seventy percent agree or strongly agree that, for new radiation oncology procedures and technologies, expert users should submit commissioning reports, quality assurance tasks, and checklists for peer review and archival preference publication. The majorities of physicists (73.5%) agree or strongly agree that they should participate in a local CME program which is procedure‐specific, in order to deliver radiation therapy with high quality and safety and to keep up with the development of new technologies. Detailed survey results are shown in Table 3.

**Table 3:** Current view of medical physicists. Responses are scored as follows: Strongly Disagree – 1, Disagree – 3, Neither Agree nor Disagree – 5, Agree – 7, Strongly Agree – 10. The reported numbers below are the arithmetic mean and standard deviation of these numbered responses.

*To what extent do you agree or disagree with the following statements*:	*Number of Respondents*	*Mean*	*SD*
Medical physicists, medical dosimetrists, radiation oncologists, and radiation therapists work together with excellent communication; this assures a record of excellent respecting to quality of treatment	1,009	7.37	2.27
There are adequate standards at the national and local level to credential and qualify personnel to perform advanced radiation oncology procedures	1,008	6.23	2.31
Medical physicists and other personnel should participate in a local mechanism in which procedure‐specific trainings, qualifications, and credentialing are recorded through the ongoing reporting of services provided	1,007	7.32	1.99
Manufacturers' training programs are completely adequate to train physicists and others to deliver radiation therapy with high quality and safety	1,007	4.39	2.14
When commissioning new special procedures, an outside procedure review by a qualified medical physicist familiar with the procedure should be standard practice in the community	1,008	7.35	1.99
When new special procedures are introduced into the community, adequate training of sufficient depth and quality is being provided at targeted forums led by expert early adopters and users	1,003	5.72	2.24
There are too few manufacturer‐supported continuing education forums that offer clinically useful material for new and emerging technologies	1,006	6.46	1.94
For new radiation oncology procedures and technologies, expert users should submit commissioning reports, quality assurance tasks, and checklists for peer review and archival preference publication	1,007	7.31	1.97
In spite of the complexity associated with IMRT, IGRT, adaptive therapy, segmentation, and gating technologies, the “Medical Event” is still a valuable concept to elucidate whether radiation therapy was performed improperly	1,007	6.88	2.04

The scope of inquiry in Table 3 indicates that measurement and demonstration of safety and quality are very complex concepts. Medical physicists agree that communication of treatment processes and procedures is key to assuring safety, that all involved in the treatment of special procedures should demonstrate continuing practice competence, and that outside review of special procedures processes by competent professionals should be a standard radiation oncology practice. First adopters of new technologies should provide detailed peer‐reviewed commissioning reports and checklists to the community in the form of archival publications in respected journals.

Medical physicists indicate there is room for improvement in the definition of national and local practice standards for special procedures. In particular, the quality and depth of manufacturer's training and the limited access to medical physicist domain experts in new technologies with respect to practice standards need systematic review and improvement. This will take cooperation between national organizations and equipment vendors to bring about a needed culture change. Targeted society forums addressing new technologies and manufacturer continuing education forums may be an important part of that culture change. Finally, the “Medical Event” has support as a concept of quality determination, despite the increasing complexity of evaluating the quality of radiation treatment delivery.

## IV. CONCLUSIONS

The 2012 ASTRO Workforce Survey mapped out the characteristics of the current radiation oncology workforce, as well as the needs and concerns of the current workforce. The survey results will be valuable in assisting us in predicting future manpower needs, providing guidance in developing future policies to meet rising demands of radiation therapy, and ensuring there is adequate manpower to provide quality and safe patient care. Medical physicists should participate in a local mechanism in which procedure‐specific trainings, qualifications, and credentialing are recorded through the ongoing reporting of services provided to ensure only Qualified Medical Physicists (QMPs)^(8)^ are providing medical physicist services.
